# The efficacy of intraoperative methylene blue enemas to assess the integrity of a colonic anastomosis

**DOI:** 10.1186/1471-2482-7-15

**Published:** 2007-08-02

**Authors:** Stanton Smith, William McGeehin, Robert A Kozol, David Giles

**Affiliations:** 1 University of Connecticut School of Medicine, Farmington, CT, USA; 2 Charlotte Hungerford Hospital, Torrington, CT, USA; 3 University of Connecticut School of Medicine, Farmington, CT, USA; 4 University Connecticut School of Medicine, Farmington, CT, USA

## Abstract

**Background:**

Intraoperative testing of colonic anastomoses is routine in assuring anastamotic integrity. We sought to determine the efficacy of the methylene blue enema (MBE) as an intraoperative test for anastomotic leaks.

**Methods:**

This study is a retrospective review of consecutive colonic operations performed from January 2001 to December 2004 in a community hospital setting by a general surgical group that uses the MBE exclusively. All operations featuring a colonic anastomosis and an intraoperative MBE were studied (n = 229). Intraoperative MBE via a rectal tube was used as the diagnostic test. Intraoperative leak (IOL) rate and clinically significant postoperative leak (POL) rate were the outcome measures.

**Results:**

The IOL rate was 4.5% for proximal anastomoses, 8% for distal anastomoses, and 7% of total anastomoses. The POL rate was 3% of anastomosis. There were no other testing methods employed. There were no POLs in cases where an IOL led to concomitant intraoperative repair. POL rate for proximal anastomosis was 0.8% and for distal 3%, for stapled 1% and hand sewn 5%.

**Conclusion:**

MBE IOL rate is comparable to published IOL rates for other methods of intraoperative testing. The MBE can be applied to proximal and distal anastomosis. Patients who were found to have an IOL, and underwent immediate repair, did not develop a clinical POL.

## Background

Colon surgery often requires a resection of part of the colon with a subsequent anastomosis to reestablish continuity. The prevalence of intraperitoneal anastomotic leak has been reported between 0.5% to 30%, but is generally between 2% and 5% in the modern literature [1]. The importance of avoiding clinically evident leaks is reflected in a mortality rate 3 to 13 times higher in the population that leaks versus those with an uncomplicated course [2,3]. Despite a long history of colonic anastomosis and associated literature, the more recent advent of the stapled anastomosis brought with it intraoperative testing.

Despite a relative paucity of literature specifically centered on intraoperative anastomosis testing, the practice of intraoperative testing of new anastomosis in the distal colon has become standard. A variety of techniques exist, with no literature comparing methods, frequency of use, efficacy or safety. While air/water testing is common, we report an experience with a less common technique, a methylene blue enema (MBE). Used as an intravenously administered urologic dye, for sentinel node biopsies, and for detecting gastric leaks, methylene blue has an established safety profile, and is readily available [4,5]. We hypothesize that the MBE will prove as efficacious and efficient in identifying anastomotic leaks intraoperatively as other accepted methods of intraoperative testing.

## Methods

### Design

We performed a retrospective review of consecutive colonic anastomoses that were performed by one surgical group that uses the MBE exclusively in a community hospital from January 2001 through December 2004. Basic demographic details were gathered, as well as details of the surgery and its results. We defined an intraoperative leak (IOL) as visualization of methylene blue dye outside of the colon (in the operative field). Postoperative leaks (POL) are defined clinically as a constellation of fever, abdominal pain/peritonitis, and leukocytosis, radiographically, or by the institution of treatment indicating that the patient had a leak (such as the creation of a diverting ostomy). Deaths were reviewed for the possibility of unrecognized POLs. Differences between intraoperative and postoperative leak rates were analyzed. All advanced statistical analysis was carried out with SPSS software, version 12.01 for Windows. The University of Connecticut Health Center and Charlotte-Hungerford Hospital IRBs approved the study.

### MBE Technique

**Figure 1:**
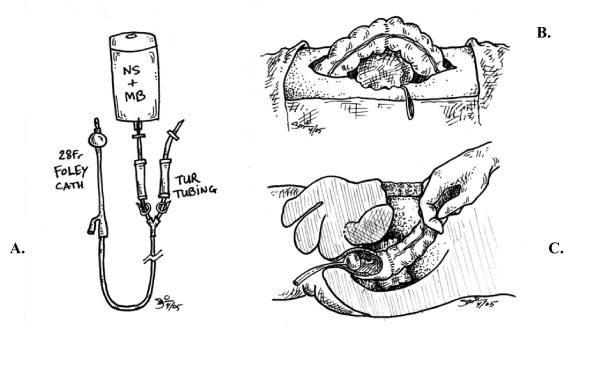
**MBE Apparatus and Method**. **A**. Apparatus for methylene blue enema. **B**. Anastomosis with gauze pads beneath: **C**. Cross sectional view (pelvis).

The apparatus for the enema includes a 28 French Foley catheter with a 30 mL balloon, tubing, and a 1 liter of normal saline containing 10 mL of 1% methylene blue dye (final concentration of 0.01%). The rectal tube is inserted after anesthesia is induced when resecting the proximal 2/3 of colon and delayed until after anastomosis is completed for the distal 1/3 of the colon and the rectum. The Foley balloon is inflated and gently withdrawn to the internal anal sphincter to prevent leakage around the balloon. The anastomosis is surrounded with clean sponges, the colon is occluded proximally and is allowed to fill as the fluid bag was raised. If a leak was present, it will be visualized as spillage of blue dye. When the surgeon is satisfied with the anastomotic integrity, the fluid bag is lowered to the floor to allow gravity drainage. This procedure could be repeated multiple times as needed. (Figure 1)

## Results

From January 2001 through December 2004, 229 patients underwent colonic resection with MBE testing. The gender was male in 49%, the mean age 61 yrs (46–87) and length of stay 6.4 days (1.7–11.1). Operations were elective 88.6% of the time, urgent 9.6%, and emergent 1.7%. The indications for surgery were known carcinoma (21%), diverticular disease (19%), polyps (17%), reversal of Hartmann's procedure (12%), colonic masses (10%), perforation (5%), colonic obstruction (2%) and other (14%). Cancer was in the final diagnosis 39% of cases.

The most common operations performed were sigmoid colectomy 36% of the time and right hemicolectomy 24%. Anastomoses were stapled 64% of the time. The operations were laparoscopic (hand-assisted) 30% of the time. Nine anastomoses were protected with an ostomy despite no IOL, and are excluded from POL and MBE test analysis. (Table 1)

**Table 1:** Operative details with MBE, IOLs, and POLs.

**Variable**	**Enema (n = 229)**	**IOL (n = 16)**	**POL (N = 7)**
Operation:	#	#	#
R hemi- and Extended R colectomy	55	3	1
Transverse	10	0	0
L hemi- and Extended L colectomy	17	2	1
Sigmoid	84	8	3
LAR	31	1	1
Hartmann's Closure	29	2	1
Other*	3	0	0

Method:	#	#	#
Open	130	9	4
Lap-assisted	68	4	3
Lap to open	31	3	0

Anastomosis type:	#	#	#
Hand sewn	83	4	4
Stapled	146	12	3

	#	#	
Diverting ostomy	15**	6	n/a

*Proximal and sigmoid colectomies (2 anastomoses) ×2, subtotal colectomy. **Including the 6 in response to IOL.

There were 16 (7%) intraoperative leaks (IOL). The IOL rate for an anastomosis proximal to the left colon was 4.5% (3/67), distal 8% (13/162). In the patients with IOLs, 12 were repaired with interrupted silk sutures, 3 had a redo anastomoses and one was not repaired (diverting ileostomy only). All repairs were tested a second time with MBE. One patient had a second IOL and required a third MBE after additional interrupted silk sutures had been placed. Five repairs were protected with a diverting ileostomy (bringing the total of diverting ileostomies in the entire study group to 16.

There were 7 postoperative leaks (POL), a POL rate of 3.3%. There were no POLs in the patients who had an IOL. The POL rate for a proximal anastomosis was 1.5% and distal 4%, for stapled anastomoses 2%, hand sewn 5%. Two POLs were diagnosed in the same admission, 4 on readmission and the seventh could not be excluded on review of a long, postoperative ICU course, which was ultimately fatal. Ages ranged from 53–82 years. Of the 7 cases with leaks, 6 were elective procedures, with one proximal anastomosis and 5 distal. Two operations were for carcinoma, 3 for diverticular disease (one colovesicular fistula) and one a Hartmann's reversal. The seventh was emergent for a lower gastrointestinal bleeding (LGIB). Of the cases with POLs, the anastomosis was stapled in 3, hand sewn in 4. Of the 6 elective procedures, operative time ranged from 42–164 minutes, estimated blood loss ranged from 50–950 mL and intraoperative urine output from 130–500 mL. The ASA class was III for three (two in class II, one in class I). All received prophylactic antibiotics and bowel preps, and required a second operation (two ileostomies, four colostomies). The seventh patient did not have a second operation as a leak was never demonstrated and the patient died. The mortality from a POL was 14%.

The sensitivity of the MBE as a test for POL is inappropriate because a false positive does not exist. The specificity of the MBE as a test for a POL is 95%. Likewise the positive predictive value (PPV) of MBE for POL is inappropriate because all IOLs resulted in intervention. The negative predictive value (NPV) of the MBE for POL was 97% (Table 2).

**Table 2:** Leak Results (for those receiving a MBE without a diverting ostomy)

	**Positive POL #**	**Negative POL #**	**Total #**
**Positive IOL**	0	10	10
**Negative IOL #**	7	197	204
**Total #**	7 (3%)	207	214

MBE ability to predict a POL: Sensitivity = 0/(0+7) = 0; Specificity = 197/(197+10) = 95%; Pos. Pred. Value = 0/(0+10) = 0; Neg. Pred. Value = 197/(197+7) = 97%

The overall 30-day mortality for all colonic anastamoses was 1% (3 of 229), and 14% (1 of 7) for those with POL. The causes of death were progressive cerebral vascular accident (CVA), aspiration with arrest and withdrawal of ventilator support in the setting of multiple system organ failure (MSOF) (the presumed POL). The average age was 71 yrs., with one operation each elective, urgent, and emergent. A right-sided anastomosis was performed in one, a left sided anastomosis in two. All were without an IOL, nor was a POL demonstrated (although presumed in one).

## Discussion

In our experience, the MBE has shown applicability to all colonic anastomoses. Compared to the literature on this topic, the results here match exactly the IOL rate for the total of other studies (Table 3) [6-13]. Our POL rate is lower, perhaps in part due to the mixed locations of our anastomoses and 31% diversion rate of those with an IOL. The MBE was utilized less frequently for right-sided anastomoses, a decision supported by the finding of no POLs in patients who had not had a previous MBE.

**Table 3:** Comparison of reported clinical leak rates by author.

**Author**	**Year**	**n (Tested)**	**Method**	**Anastomosis Location**	**IOL #(%)**	**POL #(%)**
Davies [6]	1988	33	Air	Low Anterior Resection	6 (18)	4 (12)
Gilbert [7]	1988	21*	Saline	Low vs. Low Anterior	5 (24)	1 (5)
Beard [8]	1990	73	Air	Above/Below Reflection	18 (25)	3 (4)
Pritchard [9]	1990	82**	Air	High/low Anterior	5 (6)	8 (10)
Griffith [10]	1990	60**	Air	Anterior Resection	11 (18)	2 (3)
Dixon [11]	1991	119**	Saline	Anterior Resection	5 (4)	2 (2)
Wheeler [12]	1999	102	Saline	Anterior/Low Anterior	21 (21)	7 (7)
Schmidt [13]	2002	260***	Air/MBE	Low Anterior Resection	47 (18)	27 (10)

Total		750			118 (16)	54 (7)

*Hand sewn anastomoses. **Stapled anastomoses. ***Cases from 1995–2000, stapled anastomoses.

As a diagnostic test to predict POL, the MBE had no sensitivity or PPV. It was flawless in preventing a POL in a patient with an IOL, provided of course that the IOL result was acted on. This seems intuitive, and is consistent with findings reported by Griffith [10]. Only one case report of a positive IOL not being repaired was found and that patient subsequently had a POL [6]. Because the specificity and NPV are not perfect, a negative result on a MBE does not guarantee a POL will not follow. This finding is consistent with all the studies in Table 3 and with Pritchard who suggested the intraoperative test might be misleading because in his 8 patients who had a clinical leak, all were "watertight" on intraoperative testing [9].

The small number of patients with POL precludes statistical analysis of characteristics of patients with POL (compared to those without POL). Six were elective cases, and had prophylactic antibiotics, bowel preps, and a general anesthesia component (3 with epidurals). Four patients had anemia and two others had peripheral vascular disease. Of note, six of these anastomoses were left sided. Although the precise distance from the anal verge was not always noted, an inverse relationship is well documented between level of the anastomosis and clinical leak rate [8,9,14].

This study does not offer a controlled comparison of MBE to other methods. The MBE has an advantage over air/water testing in that the bubbles in air/water testing are at times difficult to localize on the anastomosis. Conversely, with MBE, the blue staining on the gauze precisely shows the area of leakage. One disadvantage of MBE is that it cannot be used on anastomoses within 6 cm of the anal verge as the enema balloon could not be accommodated. It seems doubtful that utilizing the two methods together would be more helpful. The optimal pressure recommended for detecting intraoperative leaks with air/water testing was 25 cm H2O by Gilbert [7], and 30 cm H2O (via a manometer) with fluid enema by Wheeler [12] with apparently similar results.

The safety of this use of methylene blue appears very good. No untoward effects have been recognized in over 10 years of MBE use. Toxic if more than 4 mg/kg is acutely ingested in normal adult patient, a total of 100 mg of methylene blue is used in the liter of saline. As the colon is routinely emptied of the enema after use, a variable amount of residual will remain in the colon. Usage in the pediatric population for individuals less than 25 kg, in which the whole liter is used, potentially could be problematic. Methylene blue has also been safely used in sentinel lymph node biopsies for breast cancer, thyroid cancer and melanoma 13. At a cost of approximately $1.67 (USD) 13 per 5 mL vial, it appears relatively comparable to the cost of cleaning and operating an endoscopic apparatus such as a sigmoidoscope.

While there is unlikely to be any one holy grail, the MBE represents a simple, inexpensive and versatile method of testing colonic anastomoses. MBE is as efficacious in identifying IOLs as other accepted methods, and may be more frequently used as awareness of this technique increases.

## Authors' contributions

DG, SS, WG, RK: Study conception and design.

SS: Acquisition of data.

DG, SS: Drafting of Manuscript.

DG: Supervision.

RK: Final approval of the version to be published

## Pre-publication history

The pre-publication history for this paper can be accessed here:


